# Gene Expression Profiling in Gastric Mucosa from *Helicobacter pylori*-Infected and Uninfected Patients Undergoing Chronic Superficial Gastritis

**DOI:** 10.1371/journal.pone.0033030

**Published:** 2012-03-16

**Authors:** Ze-Min Yang, Wei-Wen Chen, Ying-Fang Wang

**Affiliations:** 1 Pi-Wei Institute, Guangzhou University of Chinese Medicine, Guangzhou, Guangdong, China; 2 School of Basic Courses, Guangdong Pharmaceutical University, Guangzhou, Guangdong, China; 3 School of Chinese Materia Medica, Guangdong Pharmaceutical University, Guangzhou, Guangdong, China; 4 E-Institute of Traditional Chinese Medicine Internal Medicine, Shanghai Municipal Education Committee, Shanghai, China; University of Hyderabad, India

## Abstract

*Helicobacter pylori* infection reprograms host gene expression and influences various cellular processes, which have been investigated by cDNA microarray using *in vitro* culture cells and *in vivo* gastric biopsies from patients of the Chronic Abdominal Complaint. To further explore the effects of *H. pylori* infection on host gene expression, we have collected the gastric antral mucosa samples from 6 untreated patients with gastroscopic and pathologic confirmation of chronic superficial gastritis. Among them three patients were infected by *H. pylori* and the other three patients were not. These samples were analyzed by a microarray chip which contains 14,112 cloned cDNAs, and microarray data were analyzed via BRB ArrayTools software and Ingenuity Pathways Analysis (IPA) website. The results showed 34 genes of 38 differentially expressed genes regulated by *H. pylori* infection had been annotated. The annotated genes were involved in protein metabolism, inflammatory and immunological reaction, signal transduction, gene transcription, trace element metabolism, and so on. The 82% of these genes (28/34) were categorized in three molecular interaction networks involved in gene expression, cancer progress, antigen presentation and inflammatory response. The expression data of the array hybridization was confirmed by quantitative real-time PCR assays. Taken together, these data indicated that *H. pylori* infection could alter cellular gene expression processes, escape host defense mechanism, increase inflammatory and immune responses, activate NF-κB and Wnt/β-catenin signaling pathway, disturb metal ion homeostasis, and induce carcinogenesis. All of these might help to explain *H. pylori* pathogenic mechanism and the gastroduodenal pathogenesis induced by *H. pylori* infection.

## Introduction

*Helicobacter pylori* (*H. pylori*) is a spiral-shaped Gram-negative bacterium that colonizes the stomach in about 50% of all humans. Even if the majority of *H. pylori*-colonized individuals remain asymptomatic, the *H. pylori* infection is the most primary cause of chronic gastritis and known to be a risk factor for peptic ulcer disease and gastric malignancy. It is also the first bacterium observed to act as a carcinogen.

The development of chronic gastritis associated with *H. pylori* infection is a multifactorial process. Both *H. pylori* and host factors influence the pathogenesis of chronic gastritis. On the *H. pylori* side, virulence factors produced by *H. pylori* not only damage directly gastric epithelial cell, but also increase inflammatory cytokine production, epithelial cell proliferation and apoptosis. Vacuolating cytotoxin(VacA) and cytotoxin-associated gene A protein (CagA) are major virulence factors. CagA is translocated into gastric epithelial cells by a type IV secretion system, encoded by the Cag-pathogenicity islands. Both phosphorylated and nonphosphorylated CagA can activate cytoskeletal reorganization and several signal transduction pathways, such as PI3A/Akt, beta-catenin and NF-kappaB (NF-κB) signaling, which promote proliferation and inflammation [1]–[2]. VacA secreted by type V-secretion system is internalized into host cells by endocytosis and influences different cellular processes, including cell vacuolation, mitochondria-dependent cell death and an increase in the permeability of gastric epithelium, inhibition of T-cell activation and proliferation, and initiation of proinflammatory response [3]. Besides, VacA also activate PI3A/Akt, MAPK and NF-κB signaling [3]–[4]. Neutrophil-activating protein (HP-NAP) secreted by *H. pylori* is also an important virulence factor due to its ability to induce neutrophils to produce reactive oxygen radicals [5]. HP-NAP is an immune modulator and is able to induce the expression of interleukin-12 (IL-12), IL-23 and IL-8 by stimulating different inflammatory cells, such as neutrophils, monocytes and dendritic cells. HP-NAP is now considered as a crucial factor in driving the Th1 inflammation in *H. pylori* infection [6]–[7]. *H. pylori* lipopolysaccharide (HP-LPS) enhances cell proliferation and inflammation via the MEK1/2-ERK1/2 mitogenactivated protein kinase cascade and Toll-like receptor (TLR) in gastric epithelial cells [8]. *H. pylori* heat-shock protein 60(HP-HSP60) induces inflammatory response via the Toll-like receptor and MAPK pathways in monocytic cells, Macrophages and gastric epithelial cells [9]–[11]. OipA, known to influence the risk of developing clinical *H. pylori*-related diseases, plays a role in *H. pylori*-induced focal adhesion kinase activation and cytoskeletal re-organization. In addition, some researches have shown other pathopoiesis factors secreted by *H. pylori* were involved in host cell inflammatory reaction [12]–[14]. Therefore, activating host inflammatory reaction and several signal transductions may be the primary pathopoiesis mechanism of *H. pylori*.

On the host side, host counteracts *H. pylori* infection by alteration in different aspects. Secretion of antibacterial substances and gastric mucosal barrier are the important defense mechanisms of the stomach to limit the proliferation of *H. pylori*. Lactoferrin inhibits bacterial growth by restricting the availability of extracellular iron ions, and Lactoferrin also has antibacterial characteristics [15]. Components of the gastric mucin can inhibit biosynthesis of the *H. pylori* wall. Neutrophils and macrophages produce large amounts of reactive oxygen species (ROS) and nitric oxide (NO) which can produce reactive nitrogen species (RNS) by reacting with O2^ ˙-^ [16]–[18]. ROS and RNS can directly kill bacteria. In addition, host inflammatory and immunological reaction to *H. pylori* increases. The chronic infection was characterized by increasing the numbers of lymphocytes, macrophages, neutrophils, mast cells and dendritic cells, and the infiltration of inflammatory cells into the sub-epithelial gastric lamina propria. A humoral immune response to *H. pylori* is elicited in nearly all *H. pylori* -infected humans. IFN-γ, TNF, IL-6, IL-7, IL-8, IL-10 and IL-18 levels are increased in the gastric mucosa [19]–[20]. Monocyte-derived human dendritic cells release cytokines and increase expression of major histocompatibility class II proteins [21]. Moreover, *H. pylori* infection can enhance cell proliferation [8], [22] as to retrieve gastric mucosa damage and apoptosis of gastric epithelial cells [18], [23]. Thus, to counteract *H. pylori* infection, host activates gene transcription involved in defense mechanism, inflammatory and immunological reaction, cell proliferation and apoptosis.

Analysis of host gene expression profiling in response to *H. pylori* infection might be one approach to better understand the role of host factors in pathogenesis. cDNA microarray has been used by many researches to investigate the alterations in host gene expression induced by *H. pylori* infection. These previous studies were based mainly on either *in vitro* culture cells or *in vivo* animal models [24]–[29]. For instance, studies in rhesus macaque and U937 cells found that *H. pylori* infection altered host expression of genes related to immune evasion, inflammatory and immune responses, signal transduction and transcription factors [24]–[25]. Gene expression profiling has also been employed to investigate human gastric mucosa or biopsy tissues infected by *H. pylori* in Europe and South America. In these studies, the samples used for gene microarrays are from those of the chronic abdominal complaints related to non-atrophic and atrophic gastritis, duodenal and gastric ulcers. And gene chips used in previous researches were mostly of low-density which had a relatively limited number of genes [30]–[32], with a recent exception in which whole genome microarray representing more than 47,000 transcripts was used to investigate the gene expression profiling in European patients infected with *H. pylori* [33]. However, rare report, especially in Asia, is on the analysis of gene expression profiling of human gastric mucosa from *H. pylori*-related chronic superficial gastritis using high-density cDNA microarray.

In the present work, chronic superficial gastritis which is a very common disease of chronic abdominal complaints in China was selected as disease mode of *H. pylori* infection, and high-density cDNA microarray including 14,112 cloned cDNAs was used for chip experiment. The gene expression profiles of *H. pylori*-infected and uninfected patients undergoing chronic superficial gastritis were analyzed by BRB ArrayTools and IPA software. Alteration of gene expression profiles was further analyzed to explore the effects of *H. pylori* infection on the host gene expression. Comparison of these profiles with published pathological literature may contribute to the identification of genes associated with *H. pylori* infection and better understanding to pathogenic mechanisms of *H. pylori* and pathophysiological mechanisms involved *H. pylori*-infected chronic superficial gastritis.

## Results

The present study was designed to identify gastric mucosa genes, biology processes and molecular interaction networks correlated with *H. pylori* infection. The cDNA microarray technology was used to explore the change in gene expression profiles between *H. pylori* infected and uninfected patients with chronic superficial gastritis. The differentially expressed genes identified by BRB ArrayTools soft were found to be related to various biology processes including protein metabolism, inflammatory and immunological reaction, signal transduction, gene transcription, trace element metabolism, and so on. By IPA Core Analyses, most of these genes were categorized in three molecular interaction networks involved in gene expression, inflammatory response and cancer progress.

### Identification of differentially expressed genes in *H. pylori*-infected and uninfected patients undergoing chronic superficial gastritis

**Figure 1:**
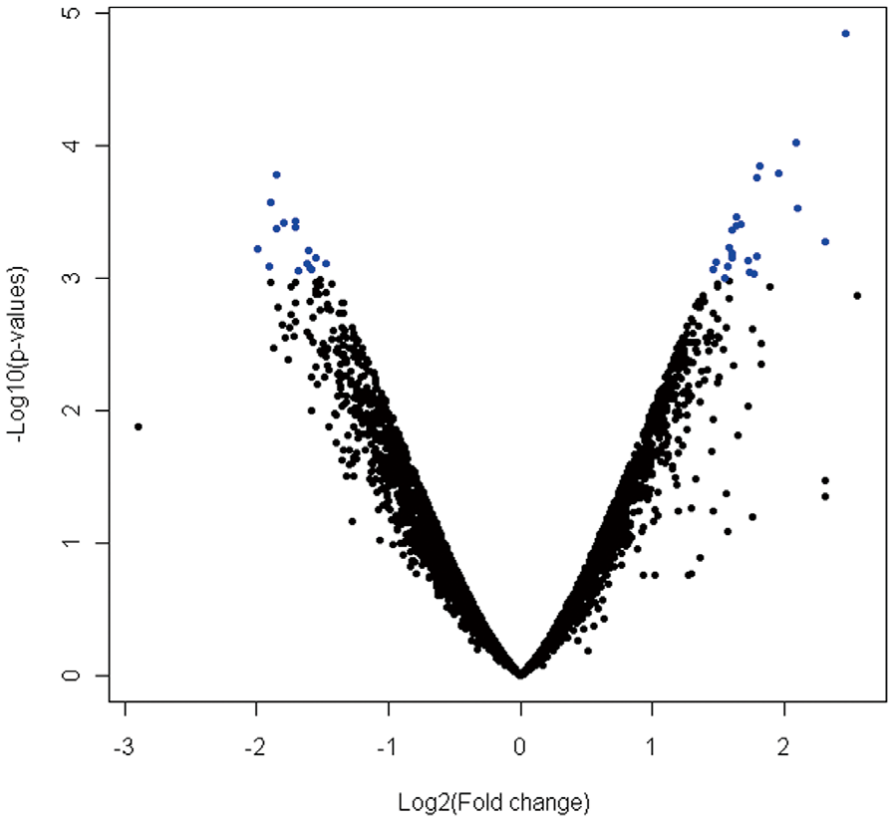
The scatterplot of differentially expressed genes in *H. pylori*-infected and uninfected patients undergoing chronic superficial gastritis. Log_2_ fold changes and their corresponding -log_10_ p-values of all genes in the microarray were taken for construction of the scatterplot. Negative values of Log_2_ fold changes indicate genes down-regulated and positive value is up-regulated. Differential expressed genes with more than 2.0 fold change and a p-value<0.001 are depicted in blue dots. All other genes in the array that were not found to be significantly altered are in black dots.

We adopted gene chips containing 14,112 cloned cDNAs to compare the gene expression profiles between *H. pylori* infected and uninfected patients with chronic superficial gastritis. The chip data were analyzed by BRB ArrayTools software. Out of all cloned cDNAs, 6,143 genes were matched with data filtering condition. Thirty-eight genes were significantly correlated with *H. pylori* infection in gastric mucosa of patients with chronic superficial gastritis, which were defined as differentially expressed genes. These thirty-eight differentially expressed genes included 23 up-regulated genes and 15 down-regulated genes. The scatterplot of differentially expressed genes was shown in Figure 1. Among 38 genes, the 34 differentially expressed genes have their genes annotation information, which are involved in protein metabolism, inflammatory and immunological reaction, signal transduction, gene transcription, trace element metabolism, and so on (Table 1).

**Table 1:** The differentially expressed genes in *H. pylori*-infected and uninfected patients undergoing chronic superficial gastritis grouped by function.

Gene name	Fold Change
**Protein metabolism**	
RPS14: ribosomal protein S14	3.448
RPS27: ribosomal protein S27	5.556
WASH1: WAS protein family homolog 1	3.846
GOLPH3: golgi phosphoprotein 3 (coat-protein)	−3.740
ASB15: ankyrin repeat and SOCS box-containing 15	−3.270
RWDD4A: RWD domain containing 4A	−2.780
RNF138: ring finger protein 138	−3.460
PSMD5: proteasome (prosome, macropain) 26S subunit, non-ATPase, 5	3.333
PSME2: proteasome (prosome, macropain) activator subunit 2 (PA28 beta)	2.778
**Cell adhesion, inflammatory and immunological reaction**	
CEACAM8: carcinoembryonic antigen-related cell adhesion molecule 8	3.226
LGALS3BP: lectin, galactoside-binding, soluble, 3 binding protein	3.448
MUC16: mucin-16	−3.220
HLA-DRB1: major histocompatibility complex, class II, DR beta 1	5.000
C5ORF53: chromosome 5 open reading frame 53	−3.270
GPX1: glutathione peroxidase 1	3.333
NOSIP: nitric oxide synthase interacting protein	2.778
**Signal transduction**	
SCGN: secretagogin, EF-hand calcium binding protein	−3.730
PHPT1: phosphohistidine phosphatase 1	3.448
MST4: serine/threonine protein kinase MST4	−3.990
**Gene transcription**	
C19ORF2: chromosome 19 open reading frame 2	−3.010
NR1D2: nuclear receptor subfamily 1, group D, member 2	-3.610
MED6: mediator complex subunit 6	2.941
ELP4: elongation protein 4 homolog (S. cerevisiae)	−3.030
HDAC7: histone deacetylase 7	3.030
ANP32B: acidic (leucine-rich) nuclear phosphoprotein 32 family, member B	2.941
TCEB2: transcription elongation factor B (SIII), polypeptide 2 (18 kDa, elongin B)	3.030
**Trace element metabolism**	
FTL: ferritin, light polypeptide	3.448
COMMD1(includes EG:150684): copper metabolism (Murr1) domain containing 1	3.125
SLC39A6: solute carrier family 39 (zinc transporter), member 6	−3.080
**Other**	
RNASEH2B: ribonuclease H2, subunit B	−3.610
FLJ35220: hypothetical protein FLJ35220	3.125
SULT1A4: sulfotransferase family, cytosolic, 1A, phenol-preferring, member 4	3.448
COX7B: cytochrome c oxidase subunit VIIb	3.030
ATP6AP2: ATPase, H+ transporting, lysosomal accessory protein 2	−2.940

### Biological processes of differentially expressed genes in *H. pylori*-infected and uninfected patients undergoing chronic superficial gastritis

#### Protein metabolism (Table 1)

In this study, we found 9 genes involved in protein metabolism. The 4 down-regulated genes were GOLPH3, ASB15, RWDD4A and RNF138, and 5 up-regulated genes were RPS14, RPS27, WASH1, PSMD5 and PSME2. Protein metabolism is a complicated process, including peptide biosynthesis, targeted transport, protein ubiquitination pathway *etc.* RPS14 and RPS27 are component of the ribosomal 40S subunit. WASH1 and GOLPH3 participate in targeted transport of peptide. WASH1 recruits and activates the Arp2/3 complex to induce actin polymerization and plays a key role in the fission of tubules that serve as transport intermediates during endosome sorting [34]. GOLPH3 is a peripheral membrane protein of the Golgi stack and has a regulatory role in Golgi trafficking, which is implicated in protein trafficking, receptor recycling, and protein glycosylation [35]–[36]. PSMD5 and PSME2 are component of proteasome and participate in ubiquitin-dependent protein degradation process. ASB15, RWDD4A and RNF138 are implicated in enzymes participating in protein ubiquitination. ASB15 is component of the ankyrin repeat and SOCS box (ASB) family and interacts with Cul5-Rbx2 to form E3 ubiquitin ligases, which plays significant roles via an ubiquitination-mediated pathway [37]. Recent research shows ASB15 regulates protein synthesis in skeletal muscle [38]. Though RWDD4A has no detailed annotation, the RWD structure significantly resembles that of ubiquitin-conjugating enzymes (E2) [39]. Thus, RWDD4A function may be related to ubiquitin-conjugating enzymes. RNF138 (also known as NARF) acts as E3 ubiquitin-protein ligase, which together with NLK, is involved in the ubiquitination and degradation of TCF/LEF. Meanwhile, RNF138 also exhibits auto-ubiquitination activity in combination with UBE2K [40].

#### Cell adhesion, inflammatory and immunological reaction (Table 1)

*H. pylori* colonized in stomach by binding of adhesin and its corresponding receptor. After *H. pylori* infection, host engenders an influx of immune effector cells and inflammatory cells into the gastric mucosa and promotes immune and inflammatory response. Thus transcripts encoding immune and inflammatory response proteins are regulated by *H. pylori* infection. We found that 7 genes could be associated with this group. Among them, CEACAM8, LGALS3BP, HLA-DRB1, GPX1 and NOSIP were up-regulated, while MUC16 and C5ORF53 were down-regulated. CEACAM8, also known as CD66b, belongs to the carcinoembryonic Ag supergene family. CD66b is an activation marker for human granulocytes and regulates adhesion and activation of human eosinophils [41]. LGALS3BP, also named as tumor-associated antigen 90K or Mac-2 BP, promotes intergrin-mediated cell adhesion and stimulates host defense against viruses and tumor cells [42]–[43]. HLA-DRB1 is required to mount an immune response. Active oxygen and NO are important mediators of inflammation. The main function of GPX1 is to protect against the damaging effect of endogenously formed hydroxyperoxides. NOSIP negatively regulates NO production [44]. MUC16 (also known as CA125) provides a protective, lubricating barrier against infectious agents at mucosal surfaces and mediates cell adhesion [45]. C5ORF53(Synonyms: IgA-inducing protein homolog, IGIP)enhances IgA secretion from B-cells stimulated via CD40 [46].

#### Signal transduction (Table 1)

SCGN, PHPT1 and MST4 join in cell signaling pathways. SCGN is a calcium-binding protein with similarities to calmodulin and calbindin-D28K and may link Ca^2+^ signalling to exocytotic processes [47]. PHPT1 catalyzes the dephosphorylation of phosphohistidine [48], while MST4 catalyzes the phosphorylation of protein serine/threonine.

#### Gene transcription (Table 1)

Several genes related to transcription regulator in gastric mucosa were regulated by *H. pylori* infection. The 3 down-regulated genes were C19ORF2, NR1D2 and ELP4, and 4 up-regulated genes of this group were MED6, HDAC7, ANP32B and TCEB2. The NR1D2 (also known as Rev-erbβ) and C19ORF2 (also known as RMP or URI) act as transcriptional corepressors and negatively modulate transcription [49]–[50]. ELP4 acts as subunit of the RNA polymerase II elongator complex, which is a histone acetyltransferase component of the RNA polymerase II (Pol II) holoenzyme and involves in transcriptional elongation. TCEB2 is a general transcription elongation factor that increases the RNA polymerase II transcription elongation [51]. HDAC7 plays a role in gene transcriptional repression by histone deacetylases [52]. ANP32B regulates gene expression by acting as a histone chaperone and inhibitor of histone acetylation [53]. MED6 is a coactivator involved in the regulated transcription of nearly all RNA polymerase II-dependent genes [54].

#### Trace element metabolism (Table 1)

FTL (ferritin, light polypeptide) acts as the storage of iron in a soluble and nontoxic state [55]. COMMD1 is a multifunctional protein and participates in regulation of the transcription factor NF-κB and control of copper metabolism [56]. SLC39A6 (Synonyms: LIV1, ZIP6) belongs to a subfamily of zinc transporters [57].

#### Other (Table 1)

Five additional genes were differentially expressed in *H. pylori* infected mucosa, which encode proteins involved in diverse intracellular functions. RNASEH2B participates in DNA replication and mediates the excision of single RNA from DNA: RNA duplexes [58]. FLJ35220 have no detailed annotation, but it may belong to the endonuclease V family according to Swiss-Prot and GO similarity analysis, and participate in DNA repair in response to DNA damage stimulus. SULT1A4 (or SULT1A3) catalyzes the sulfate conjugation of phenolic monoamines (neurotransmitters such as dopamine, norepinephrine and serotonin), phenolic and catechol drugs [59]. COX7B is the terminal component of the mitochondrial respiratory chain and catalyzes the electron transfer from reduced cytochrome c to oxygen. ATP6AP2 (Synonyms: Renin receptor) plays a role in the renin-angiotensin system (RAS) [60].

### Cluster analysis of differentially expressed genes

**Figure 2:**
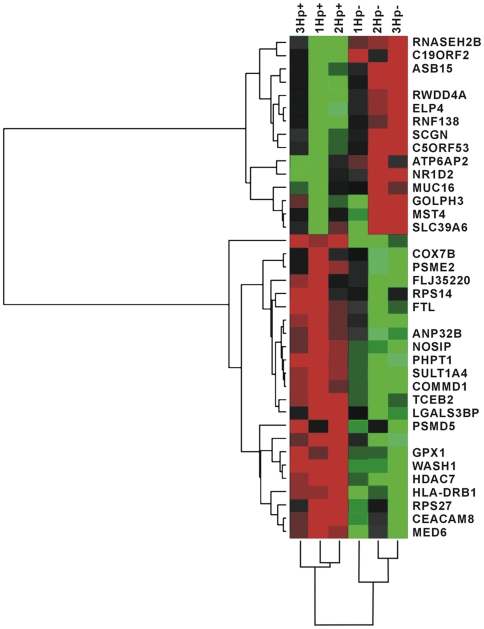
Cluster diagram of differentially expressed genes in *H. pylori*-infected and uninfected patients undergoing chronic superficial gastritis. Each column represented one sample and each row represented one gene. Red indicated higher expression and green indicated lower expression. Blank in the row indicated that the differentially expressed UniGene ID had not been annotated.

We performed cluster analysis for the samples from six patients of chronic superficial gastritis and 38 differentially expressed genes using BRB ArrayTools software. For samples, the patients were divided into two clusters of *H. pylori* infected and uninfected. For genes, down-regulated genes were clustered into two groups with C5ORF53, SCGN, RNF138, ELP4, RWDD4A, ASB15, C19ORF2 and RNASEH2B in one group, while ATP6AP2, NR1D2, MUC16, GOLPH3, MST4 and SLC39A6 in the other group; up-regulated genes were also clustered into two groups. PSMD5, GPX1, WASH1, HDAC7, HLA-DRB1, RPS27, CEACAM8 and MED6 were clustered into one group, while the rest formed the other group (Figure 2).

### Network analysis of differentially expressed genes interaction

**Figure 3:**
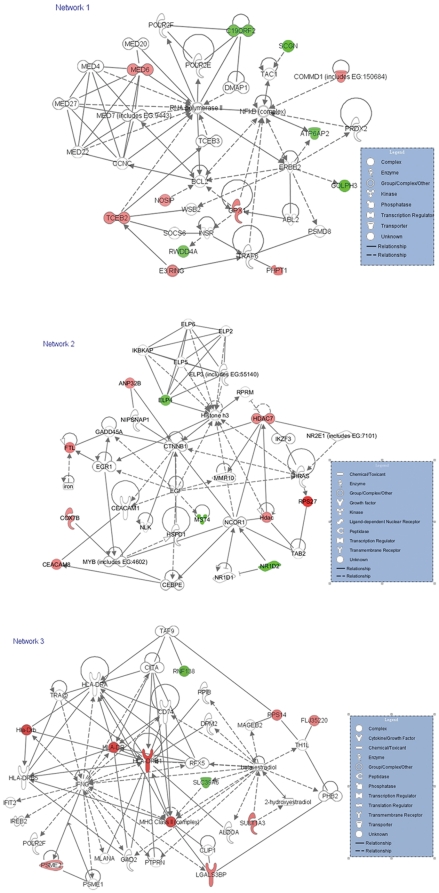
IPA network analysis of molecule interactions. The focus genes differentially expressed in this study were indicated by red symbols (up-regulated) and green symbols (down-regulated). The non-focus genes were not shown to be significant difference by the microarray analysis, and placed in the network by the Ingenuity software as intermediate molecules which were denoted by open symbols. Whereas symbols represented functional categories of the molecules are listed in the legend.

To interpret differentially expressed genes of *H. pylori* infection in the context of biological processes, pathways and networks, IPA Core Analyses were conducted and three high scoring networks (score>15) including 82% (28/34) of differentially expressed genes were identified (Figure 3). These scores, derived from *p* values, indicated the likelihood of the focus genes belonging to a network versus those obtained by chance alone, thereby eliminating the probability of their occurrence in a network to be due to noise. The network 1 with the highest score (score = 25) is comprised of gene expression, small molecule biochemistry and cancer. Other subsequent networks included network 2 (score = 19) of gene expression, cellular development, cellular growth and proliferation, and network 3 (score = 16) of antigen presentation, inflammatory response, dermatological diseases and conditions. These results implied that the altered genes were distributed among diverse networks that could be expected to the various influence on patients by *H. pylori* infection. Meanwhile, the significant (*p*<0.05) “molecular and cellular function” in IPA software was comprised of antigen presentation, cell death, gene expression, molecular transport and cellular development, while protein ubiquitination pathway was the most significant (*p* = 0.0124) “top canonical pathway” altered by *H. pylori* infection in chronic superficial gastritis.

Figure 3 showed the genes interaction in associated network. Two of the key central points in network 1 were RNA polymeraseII and NF-κB (complex). C19ORF2, MED6 and TCEB2 directly regulated RNA polymerase II, while COMMD1, GPX1 and ATP6AP2 directly interacted with NF-κB. NOSIP and GOLPH3 indirectly interacted with RNA polymerase II and NF-κB, via BCL2 and ERBB2, respectively. Besides, SCGN and PHPT1 indirectly regulated NF-κB via TAC1 and TRAF6, respectively. CTNNB1, catenin (cadherin-associated protein), beta 1, was an important central point in network 2. HDAC7 and ANP32B directly interacted with CTNNB1, while FTL, CEACAM8, MST4, NR1D2 and RPS27 indirectly interacted with CTNNB1, by GADD45A, CEACAM1, EGF, NCOR1 and HRAS, respectively. One of the important central points in network 3 was MHC Class II (complex). HLA-DRB1, SLC39A6 and LGALS3BP had direct interaction with MHC Class II. PSME2 and SULT1A3 were indirectly associated with MHC Class II by IFNG (interferon, gamma) and beta-estradiol, which were also important central points in network 3.

### SYBR green quantitative real-time PCR confirmation of selected genes

SYBR green quantitative real-time PCR was used to confirm expression data obtained for 4 of the under- and overexpressed genes detected by microarray analysis (Table 2). There was concordance between the microarray data and the real-time PCR data in all cases. However, microarray results overestimated the fold-change found by PCR.

**Table 2:** SYBR green quantitative real-time PCR confirmation of selected genes.

Gene	Microarray fold charge	Real-time PCR fold charge
HLA-DRB1	5.000	1.079
SCGN	−3.730	−1.319
COX7B	3.030	1.149
SULT1A4	3.448	1.034

## Discussion

Here, we reported the microarray results of gene expression profiling in gastric antral mucosa from patients of chronic superficial gastritis infected *H. pylori* and uninfected. Our results demonstrated that *H. pylori* infection regulated expression of genes related to protein metabolism, inflammatory and immunological reaction, signal transduction, gene transcription, trace element metabolism, and so on.

By present, most of the genes regulated by *H. pylori* infection were identified by traditional techniques such as Northern blot analysis or reverse transcriptase-quantitative polymerase chain reaction (RT-qPCR). With these techniques, one or a limited number of target genes could be selected in one experiment, which usually made, the experiments tended to validate or disprove specific hypotheses, but not lead to discovery of unexpected differentially expressed genes. However, cDNA microarray technology allowed researchers not only to study tens of thousands of genes simultaneously, but also to identify unexpected genes, which should be potently beneficial to understand pathogenic mechanism of *H. pylori*. Nevertheless, how to readout great magnanimity data produced by chip experiment is still a puzzle of this methodology. To interpret the data, there are two important works, accurately identifying the genes that were differentially expressed among groups of specimens collected from different types of tissues, and utilizing these genes to elucidate pathogenic mechanism of *H. pylori*. BRB ArrayTools is a comprehensive software developed by professional biostatisticians experienced in the design and analysis of cDNA microarray studies, which is widely recognized as the most statistically sound package available for the analysis of cDNA microarray data. In our present study, we utilized BRB ArrayTools to identify differentially expressed genes and cluster for these genes and samples. To increase data accuracy, the data filters were done before class comparison analysis, including spot filers, normalization and gene filters. Then setting of *p* value (*p*<0.001) and fold charge (exceeding 2.0) were utilized to decrease the false-positive significant rate [61]. IPA, the Ingenuity® Knowledge Base, is a repository of biological interactions and functional annotations created from millions of individually modeled relationships between proteins, genes, metabolites, complexes *etc*. These modeled relationships include rich contextual details, link to the original article, and are manually reviewed for accuracy. IPA has been broadly adopted and utilized by the life science research community. In this study, we utilized IPA to annotate gene bio-function and construct interaction network for differentially expressed genes. Thus, combining the superiorities of BRB ArrayTools and IPA with published literatures about *H. pylori* could be a better approach to understand its pathogenic mechanism in analysis of cDNA microarray.

*H. pylori* and its products/virulence factors enter the host cells, either by a type IV secretion system directly injecting into the cytoplasm (e.g. CagA), or by endocytosis mechanism being internalized into the host cell (e.g. VacA, LPS, Urease). Moreover, VacA-induced cell vacuolization is involved in endosome formation and sorting. The present study showed WASH1 which regulated endosome shape and trafficking by influencing actin polymerization [34], was up-regulated. After *H. pylori* virulence factors enter gastric epithelium, they accumulate in particle-rich cytoplasmic structure (PaCS) which arises in ribosome-rich cytoplasm. In addition to colocalization of VacA, CagA and urease, the PaCS concentrates outer membrane proteins with NOD1 receptor and ubiquitin-proteasome system (UPS) components, including ubiquitin-activating enzyme E1, polyubiquitinated proteins, and proteasome components [62]. NOD1 is a selective *H. pylori* receptor which responds to the bacterium or its virulence factors by releasing cytokines and chemokines. The UPS is the major pathway of non-lysosomal degradation of various cellular proteins. Recent study showed protein ubiquitylation had a key role in regulating immune responses [63]. Thus, PaCS may have a role in bacterial recognition and handling, and may modulate the activity of toxins/virulence factors and induce pertinent immune responses, especially through immunoproteasome [62]. In present study, the two ribosome proteins of RPS14 and RPS27, and two proteasome components of PSMD5 and PSME2 were up-regulated. These results showed that *H. pylori* infection might stimulate PaCS formation. In addition, ASB15, RNF138 and RWDD4A are involved in ubiquitylation modification which has a key role in regulating immune responses [63]. The three genes were down-regulated in our study. ASB15, participating in forming E3 ubiquitin ligases [37], is a component of the ankyrin repeat and SOCS box (ASB) family which belongs to the suppressor of cytokine signaling (SOCS) box protein superfamily [38]. The ankyrin repeats can bind to the RHD of NF-κB, masking its nuclear localization sequence, thereby retaining NF-κB in the cytoplasm and inhibiting NF-κB activation [63]. RNF138 (NARF) is a RING-containing E3 ubiquitin-protein ligase which has vital role in peripheral T-cell tolerance and inhibition of T-cell activation [63]. Moreover, RNF138 suppresses the Wnt/beta-catenin signaling [40]. The down-regulation of ASB15 and RNF138 in the present study indicated *H. pylori* infection could promote host inflammatory and immune response. RWDD4A has not been annotated and its relation to *H. pylori* infection needs be further studied. Taken together, these results demonstrated that *H. pylori* infection might stimulate PaCS formation and subsequently induce host various cellular process including inflammatory and immune responses, and signal transduction.

The prominent features of *H. pylori*-related chronic gastritis are persistent colonization of *H. pylori* and persistently enhanced inflammation with increased inflammatory cell infiltration in the local gastric mucosa. Therefore, *H. pylori* must first evolve various tricks to escape from host antimicrobial defense mechanism in order to persistently colonize in stomach. Our cDNA microarray data also provided some evidences in these aspects. MUC16, which provides a protective, lubricating barrier against infectious agents at mucosal surfaces [45], was found down-regulated by *H. pylori*. Immunoglobulin A (IgA) is a major component of the local immunity of the stomach mucosa, which plays important role in defending against pathogens invasion and maintaining gut homeostasis [64]–[65]. C5ORF53, enhances IgA secretion [46], was down-regulated by *H. pylori*. NO and ROS are important mediators of inflammation, which can directly kill bacterium. As host antimicrobial defense mechanism, ROS and NO produced by macrophages and neutrophils were induced by *H. pylori* infection [18]. However, the NOSIP and GPX1 which could inhibit NO and ROS production, respectively [44], were up-regulated by *H. pylori* in the present study. These results showed that *H. pylori* could escape the host defense mechanism by regulating related gene expression so as to persistently colonize within gastric mucosa, which was confirmed by some other chip experiment [25]. In addition, *H. pylori* and its products/virulence factors induce inflammation and immunity reactions after infecting the host cells. Several microarrays research results revealed that *H. pylori* infection significantly correlates with overexpression of MHC class II antigen-presenting genes, ubiquitin-D, CXCL-2 and -13, CCL18, and VCAM-1 genes [30], [32]–[33]. In the our present study, cell adhesion molecule CEACAM8 and MHC class II antigen-presenting gene HLA-DRB1 were found up-regulated by *H. pylori* infection, while cell chemokines or their receptors displayed no difference. The difference of our results may be due to the sample numbers and ethnical difference. Moreover, we found that LGALS3BP and SLC39A6 directly interact with MHC Class II complex in network 3 (Figure 3). Down-regulated SLC39A6 (or Zip6) can inhibit the expression of MHC class II and costimulatory molecules [66]. Thus, LGALS3BP and SLC39A6 increased host immune responses by regulating MHC Class II. Together, these results illustrated *H. pylori* could escape the host defense mechanism, as well as increase host immune and inflammatory responses.

Upon infection, *H. pylori* activates multiple intracellular pathways in epithelial cells, such as MAPK, NF-κB, Wnt/β-catenin, PI3K pathways and signal transducers [67]–[68], [4], [13]. The Wnt/Wingless signaling transduction pathway plays an important role in both embryonic development and tumorigenesis. Beta-Catenin is a key component of the Wnt signaling pathway. In the present study, beta-Catenin (CTNNB1) was an important central point in network 2 (Figure 3), which indirectly or directly interacts with several genes and most of these genes were up-regulated by *H. pylori*. In addition, RNF138, which negatively modulates Wnt/beta-catenin signaling pathway [40], was down-regulated by *H. pylori*. These results provided some evidences for *H. pylori* activating Wnt/β-catenin. NF-κB is a critical regulator of multiple biological functions including stress, injury, inflammation, innate immunity, and suppression of apoptosis [69]. The target of activated NF-κB includes the genes encoding proinflammatory cytokines, chemokines, anti-apoptotic proteins, adhesion molecules *etc*. NF-κB activation is not only induced by bacterial virulence factor/toxins (e.g. LPS, cagA, vacA), but also by pro-apoptotic and necrotic stimuli (ROS, RNS) [70]–[72]. Several studies indicated that *H. pylori*-related gastric inflammation and cancer were associated with increased NF-κB activation [23], [67], [4], [13]. In the present study, NF-κB was an important central point in network 1(Figure 3). COMMD1 and GPX1 directly activated NF-κB, while SCGN, NOSIP and PHPT1 indirectly activated NF-κB, by TAC1, BCL2 and TRAF6, respectively. In addition, Ub/proteasome pathway was also an important regulatory mechanism for NF-κB signaling, which can catalyze the proteolytic processing of NF-κB precursor and IκB. Thus, increased Ub/proteasome activities promoted NF-κB activation [73]. As previous described, PSMD5 and PSME2 composed of proteasome were up-regulated, and ASB15 participating in ubiquitylation modification, which inhibits NF-κB activation and cytokine signaling, was down-regulated in this study. These results showed *H. pylori* infection might activate NF-κB by altering related gene expression and Ub/proteasome pathway. Furthermore, some other genes involved in calcium binding and protein phosphorylation/dephosphorylation in signal transduction were altered by *H. pylori* infection. In summary, these results indicated *H. pylori* infection activated various signal transduction including Wnt/β-catenin and NF-κB.

After infection, pathogens reprogram host gene expression. In eukaryotic cells, genetic reprogramming is induced by the concerted activation/repression of transcription factors and various histone modifications that control DNA accessibility in chromatin. *H. pylori* infection induced expression of transcription factors which bound RNA polymerase II to regulate downstream gene expression [28], [67], [74]. In current study, down-regulated C19ORF2 negatively modulated transcription, while up-regulated MED6 and TCEB2 participated in transcriptional activation and elongation respectively. These three genes directly interacted with RNA polymerase II in network 1(Figure 3). NR1D2 regulated transcription as a transcriptional repressor, which was down-regulated by *H. pylori*. These results suggested *H. pylori* infection increased gene transcription process. In addition, histone modifications are critical in regulating gene expression, cell cycle, cell proliferation, and development. Recent studies have shown that histone remodeling or modification provoked by bacterial and viral pathogens is one important mechanism of regulation of immune response during infection [75]–[76]. Acetylation is one of the important approaches in histone modifications. The acetylation and deacetylation of key lysine residues of histone H3 and H4 are controlled by histone acetyltransferases and histone deacetylases (HDACs). Hamon MA group reported that listeriolysin O secreted by *Listeria monocytogenes* induced a dramatic deacetylation of histone H4 during early phases of infection and similar effect was observed in other toxins of the same family [77]. The decreased levels of histone modifications correlate with a reduced transcriptional activity of a subset of host genes, including key immunity genes [77]. Song-Ze Ding also discovered that histone H3 lysine 23 acetylation decreased during *H. pylori* infection in a cagPAI-dependent manner in AGS cells [78]. In the present study, down-regulated ELP4 is a histone acetyltransferase component of the RNA polymerase II (Pol II) holoenzyme, and is involved in chromatin remodeling and acetylation of histones H3 and H4 [79]. Up-regulated ANP32B and HDAC7 inhibit acetylation of histones [52]–[53]. These results suggest that *H. pylori* might alter histone modification by decreasing the acetylation status and affect host gene expression. However, the imcompatible results of transcription factors and histone modification remain to be clarified.

Intracellular metal ion homeostasis is necessary for almost all living organisms. Researches showed that *H. pylori* infection altered the metabolism of the trace elements including zinc and copper, especially iron, which acted as cofactor of many enzymes [80]–[81]. Iron is essential for *H. pylori* infecting and surviving in host cells. Since *H. pylori* can use different iron sources in human including lactoferrin, heme group and hemoglobin, *H. pylori* infection causes human iron deficiency [82]. Zinc is essential for the function of many enzymes and transcription factors. Zinc deficiency results in defects in innate and acquired immune responses. Research results showed SLC39A6 (or Zip6), a zinc transporter whose expression was reduced by *H. pylori* infection, inhibited the expression of MHC class II and costimulatory molecules [66]. In the present study, FTL, COMMD1 and SLC39A6, which regulated the iron, copper and zinc homeostasis and transport respectively, were altered by *H. pylori* infection.

Apart from the influence on host cells, *H. pylori* are important carcinogenic factor. Researches showed that the mechanistic link between *H. pylori* infection and gastric carcinoma was involved in oxygen free radical-induced DNA damage [83]–[84]. In general, ROS production is byproducts of energy metabolic processes in the mitochondrial electron transport chain. Thus, excessive energy metabolic might increase ROS production. Although ROS/RNS functions as antimicrobial after *H. pylori* infection, but excessive ROS/RNS production correlated well with histological mucosal damage [18]. In the present study, COX7B, which is the terminal component of the mitochondrial respiratory chain, was up-regulated by *H. pylori* infection. Moreover, down-regulated RNASEH2B participates in DNA replication, while up-regulated FLJ35220 participates in DNA repair. These results demonstrated that *H. pylori* infection increased ROS-induced DNA damage. Meanwhile, we found that several genes involved in cancer were altered by *H. pylori* infection. The genes categorized into “cancer” group by IPA biofunctions analysis were GPX1, HDAC7, PSME2, MED6, GOLPH3 and MST4 (p<0.05). The recent study indicated that LGALS3BP [43], MUC16 [50], SCGN [85] and SLC39A6 [86] had abnormal expression in various cancer. Most of these genes were clustered to two groups in Cluster diagram (Figure 2). All of these provided some evidences for *H. pylori* as a carcinogenic factor.

In addition, the 3 patients infected by *H. pylori* and 3 patients uninfected were from *Pi-Wei* dampnese-heat syndrome and *Pi*-deficiency syndrome classified by Traditional Chinese Medicine diagnosis method in our previous study, respectively [87]–[88]. They can be divided into two clusters of *H. pylori* infected and uninfected by clustering analysis of BRB ArrayTools based on differentially expressed genes. For this result, cDNA microarray technology may be useful to typing of chronic superficial gastritis based on symptom or etiologic agent.

In summary, although the sample number used in our cDNA microarray study was limited, we found several interesting categories of genes in gastric antral mucosa of *H. pylori*-infected and uninfected patients undergoing chronic superficial gastritis. These genes differentially expressed by *H. pylori* infection could be related to protein metabolism, inflammatory and immunological reaction, signal transduction, gene transcription, trace element metabolism, and so on. Combined the annotation and interaction network analysis of BRB ArrayTools and IPA software for differentially expressed genes, these data demonstrated that *H. pylori* infection could alter cellular gene expression processes, escape host defense mechanism, increase inflammatory and immune responses, activate NF-κB and Wnt/β-catenin signaling pathway, disturb metal ion homeostasis, and induce carcinogenesis. Further studies needs to be done, including collecting larger numbers of patient samples and verifying functional significance of these selected genes.

## Materials and Methods

### Data origin and process

The study data were from 3 of 6 dual channel chip data which were used to analyze gene expression profiling on chronic superficial gastritis in our previous study [87]–[88]. In this study, the 3 dual channel chip data were taken from 3 patients infected by *H. pylori* and 3 patients uninfected, which were selected to carry out bioinformatics analysis and explore the pathogenic mechanisms of *H. pylori* infection. The chip data has been stored in GEO (http://www.ncbi.nlm.nih.gov//geo) (accession numbers: GSE30042). Briefly, the six patients of chronic superficial gastritis were collected from the First Affiliated Hospital of Guangzhou University of Chinese Medicine from January to December in 2005. These patients were undergoing routine endoscopic evaluation of chronic abdominal complaints and final diagnosis of patients was confirmed by gastroscopic and pathologic examinations of gastric mucosal biopsy. Patients with chronic atrophic gastritis, gastric or duodenal ulcer and other organic diseases were excluded from the study. All patients signed the informed consent approved by the Academic Ethics Committee of Guangzhou University of Chinese Medicine. The presence of *H.pylori* infection was assessed by a positive rapid urease test. RNA extracted from gastric antrum of patients was used in cDNA microarray experiment. The cDNA microarray (BiostarH-140s) was designed by Shanghai BioStar Genechip Inc., which contained 14,112 different cloned cDNAs representing near a half of global human expressed genes and including the related genes of inflammation and immunity, apoptosis, tumorigenesis, growth and differentiation, cytoskeleton, signaling pathway, gene expression, ion channel *etc*. Samples of *H.pylori* infected were labeled by fluorescent probe Cy3, while those of uninfected by Cy5. After hybridization, the cDNA microarray was scanned and exported as image by ScanArray4000 scanner. The Spreadsheets of the dual channel chip data were obtained by Image Reader GenePix Pro 3.0 software, which included the Cy5 and Cy3 signal intensity values of all gene spots.

### Differentially expressed genes filtering

These gene spots, in which Cy5 and Cy3 signal intensity values exceeded 200 or one of them exceeded 800, and the ratio of Cy5 and Cy3 was between 0.1–10.0, were used to calculate the natural logarithm for their ratios of Cy5 and Cy3 (*r* = lnCy5/Cy3). Subsequently, the mean value (R) of all *r* value was obtained. All Cy3 signal intensity value was corrected by product of Cy3 and 1/R to decrease systemic error of Cy5 and Cy3 fluorescently labeled system. The corrected Cy3 and Cy5 signal intensity values were uploaded to BRB ArrayTools 4.1.0 Beta2 software to analyze. Intensity filers excluded the spot in which both intensities are below 200. If only one intensity was below 200, this intensity was increased to 200. A log base 2 transformation was applied to intensities before the arrays were normalized by using median over entire array. Gene filters were used to exclude corresponding spots for an entire gene from all arrays. Exclude a gene under any of the following conditions: minimum fold-change less than 20% of expression data values have at least a 1.5-fold change in either direction from the gene's median value and percent missing exceeded 50%. Filtered data were used to class comparison between groups of arrays, which was used to identify differentially expressed gene among classes of samples. The patients of *H. pylori* uninfected chronic superficial gastritis were used for the baseline gene expression. Statistics analysis was performed by paired *t*-test with random variance model and the found gene list was determined by significance at 0.001 levels of univariate tests. Negatively reciprocal transformation was done for the genes with fold change of expression less than 1. The genes which matched *p*<0.001 and fold change exceeded 2 were defined as differentially expressed genes.

### Cluster and network analysis of differentially expressed genes

Hierarchical clustering of genes and samples for differentially expressed genes were performed by BRB ArrayTools software. Analysis settings included “center the genes”, “one minus correlation metric for genes” or “centered correlation for samples”, and “average linkage”. Meanwhile, these differentially expressed genes containing the gene identifiers along with the corresponding fold changes were uploaded into the Ingenuity Pathways Analysis (IPA) website (version 8.6) (www.ingenuity.com) to annotate bio-functions, construct and visualize molecular interaction networks [89]–[90]. Briefly, the Ingenuity Knowledge Base contains information from scientific publications regarding direct and indirect relationships between genes and proteins. Each identifier was mapped to its corresponding gene object in the Ingenuity knowledge base. These genes, called focus genes, were overlaid onto a global molecular network in the Ingenuity knowledge base. Networks of these focus genes were then algorithmically generated based on their connectivity. Details and technical requirements are available in the IPA Web site (http://www.ingenuity.com/products/pathways_analysis.html) and in the supplemental methods in the online appendix.

### SYBR green quantitative real-time PCR confirmation

SYBR green quantitative real-time PCR was used to confirm the expression of *HLA-DRB1, SCGN, COX7B* and *SULT1A4*. A series of primers (Table 3) were designed using ABI Primer Express software. Reverse transcription was performed using PrimerScript RT reagent Kit and protocol (TaKaRa) in a ABI 9700 thermocycler. Real-time PCR was performed using SYBR Green Realtime PCR Master Mix (TaKaRa) in ABI 7500 FAST Sequence Detection System. Typical profile times used were: initial step, 95°C, 30 second, 95°C 5 s and 60°C 34 s, 40 cycles. *GAPDH* was used as an internal control for expression independent sample-to-sample variability. Relative gene expressions were determined from the obtained Ct values and using the 2^-ΔΔCt^ method.

**Table 3:** Primers used to SYBR green quantitative real-time PCR confirmation of selected genes.

Gene	Forward primer	Reverse primer	Product size(bp)
HLA-DRB1	GCACCCAAGCGTGACAAGCC	AGATGAACAGCCCGGCCCCA	131
SCGN	CCGGTGCCTGGTCTTCGTCG	ATCCGCATCAAAGCGCTGCCA	109
COX7B	AGCGAATTGGCACCAAAGCAGCA	CTGGTGGCTCTGCCTTGCCAT	138
SULT1A4	GCCGCACCCACCCTGTTCTC	TGAGCTCCTGGGGGACGGTG	195
